# Diagnosis of *Mycobacterium tuberculosis* Septic Shock in Patients With Anti-synthetase Syndrome Based on Next-Generation Sequencing: A Case Report and Literature Review

**DOI:** 10.3389/fmed.2021.675041

**Published:** 2021-07-01

**Authors:** Limin Sun, Ziyue Yang, Fei Yang, Zhenhua Wang, Hongqiang Li, Huifen Wang, Tongwen Sun

**Affiliations:** ^1^General Intensive Care Unit, Zhengzhou Key Laboratory of Sepsis, Henan Key Laboratory of Critical Care Medicine, The First Affiliated Hospital of Zhengzhou University, Zhengzhou, China; ^2^Department of Infectious Diseases, The First Affiliated Hospital of Zhengzhou University, Zhengzhou, China; ^3^Gene Hospital of Henan Province, Precision Medicine Center, The First Affiliated Hospital of Zhengzhou University, Zhengzhou, China

**Keywords:** *Mycobacterium tuberculosis*, sepsis, septic shock, intensive care unit, bloodstream infection

## Abstract

A 51-year-old woman was transferred to the intensive care unit with such symptoms as fever, swollen left knee joint, pain and hypotension. After preliminary evaluation, she was diagnosed as suffering acute suppurative arthritis and septic shock. Then, she was rescued and prescribed to receive treatment with broad-spectrum antibiotics. However, there was no source of infection identified except for the knee joint. The bacterial and fungal cultures of blood samples and articular effusion were shown to be negative, while the results obtained from the next-generation sequencing of blood and articular effusion revealed that *Mycobacterium tuberculosis* was positive. The patient was then put on five combinations of anti-tuberculosis therapeutic treatment. Nevertheless, despite the active anti-tuberculosis treatment put in place, her general condition still deteriorated progressively. As the level of her bilirubin continued to rise, further treatment was affected, which prompted the change made to the anti-tuberculosis treatment program. Her clinical condition continued to deteriorate, which led to the development of unstable vital signs and the multiple organ dysfunction syndrome. In spite of our best efforts to save her life, the patient still ended up with death.

## Introduction

Anti-synthetase syndrome (ASS) is referred to as a clinical classification of idiopathic inflammatory myopathy, which is mainly treated with hormones and immunosuppressants (1). The disease requires long cycle and low immunity during treatment, which makes it easy for infection to cause various complications (2). For clinicians, it is very difficult to distinguish between different types of pathogens that could cause infection, and most of them are acute and critical. Therefore, it is essential to identify what type the pathogens fall into and carry out early targeted anti-infective treatment (3). At present, there is a low positive rate of bacterial culture commonly used in clinical practice, and it requires a large amount of time, which hinders clinicians from carrying out targeted anti-infective treatment in a timely manner (4). As a novel diagnostic method, next-generation sequencing (NGS) technology can be applied to provide etiological basis at the genetic level and enable the identification of pathogens within 24 h (5). In addition, it can support the effort to make etiological diagnosis of difficult infection, for the early start of anti-infective treatment, which demonstrates significant clinical value. In this study, a dermatomyositis patient is reported who got admitted to the intensive care unit because of various clinical symptoms including knee joint redness, swelling, heat and pain, high fever, septic shock and multiple organ failure. As suggested by NGS, the pathogen was *Mycobacterium tuberculosis*. The late diagnosis rendered the prognosis of the patient's poor.

## Case Presentation

A 51-year-old woman with a one-month history of redness and swollen left knee joint was transferred to our general intensive care unit (ICU) due to persistent high fever (up to 39.5°C) and sudden hypotension. After being diagnosed with dermatomyositis 2 years ago, she continued medication treatment on prednisone tablets with a 25 mg daily dose and cyclosporine capsule with a 75 mg dose twice per day. Last month, the results of blood culture and bacterial culture of joint cavity pus as obtained from other medical institutions were shown to be negative, with no special microbiological examination performed.

**Figure 1:**
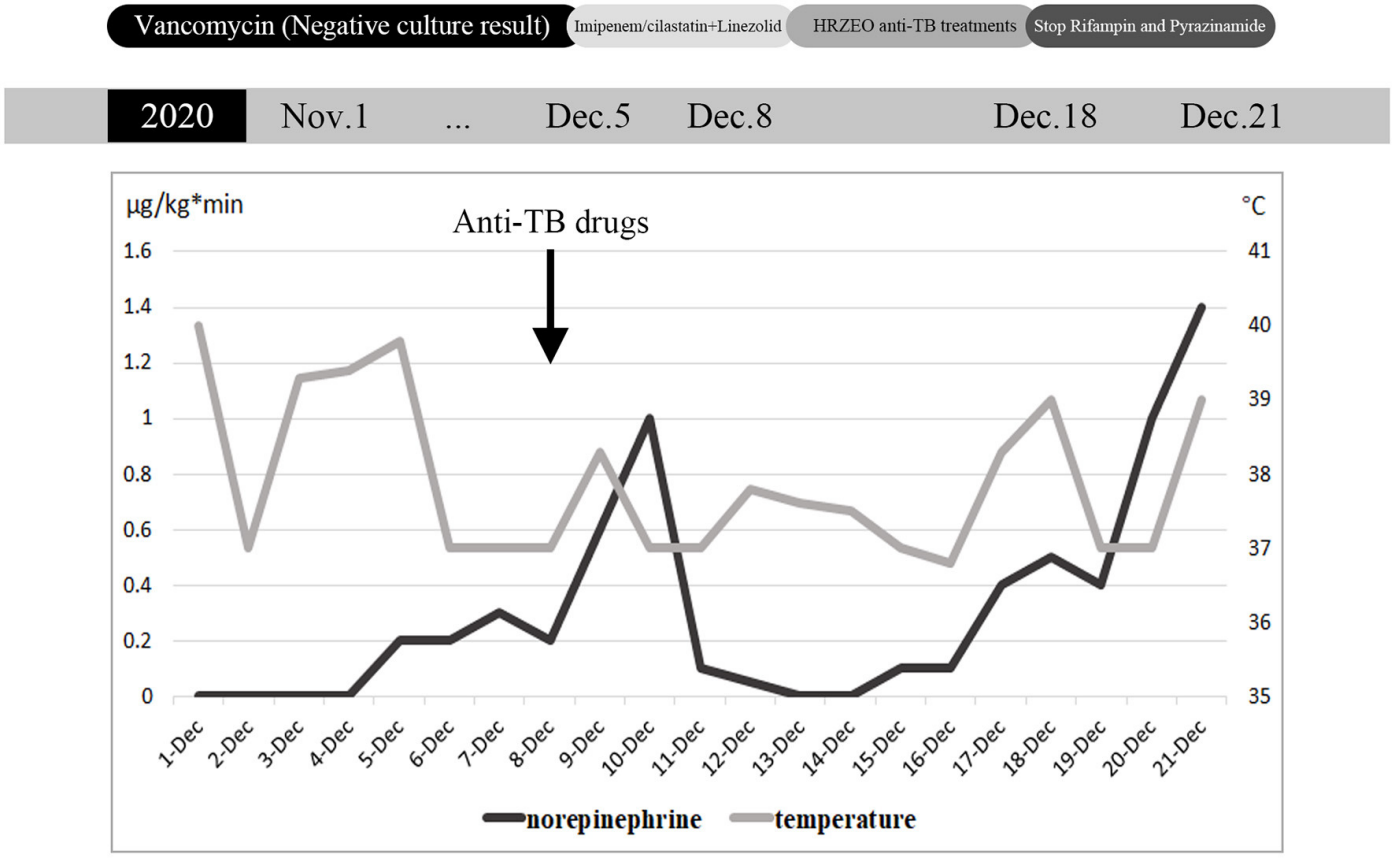
Daily maximum body temperature and maximum norepinephrine pump speed during hospitalization, and antibiotic adjustment process.

When the patient visited the rheumatology department for appointment with a doctor, she coughed up sputum intermittently and her body temperature reached up to 40°C. Physical examination revealed that the left knee was red and swollen with positive floating patellar test, along with red rash in the V area of face and neck. The right dark yellow pleural effusion displayed exudate and negative bacterial culture. Vancomycin was thus prescribed to reduce infection, and 20 mg of methylprednisolone was applied twice a day. Four days later, she was transferred to ICU due to hypotension (the detailed clinical course is shown in Figure 1). The patient exhibited low blood pressure and high heart rate, which was accompanied by elevated procalcitonin and multiple organ failure. Broad-spectrum anti-infective therapy (imipenem/cilastatin + linezolid) was initiated within the first hour of highly suspected septic shock. ECG suggested cardiac infarction and high myocardial enzyme index. In order to rule out the occurrence of acute cardiovascular event, no infarction was detected immediately after coronary angiography. Given the EF value of 30% for the patient, septic heart disease was suspected. At the same time, the patient suffered acute liver injury, acute renal injury and thrombocytopenia.

**Figure 2:**
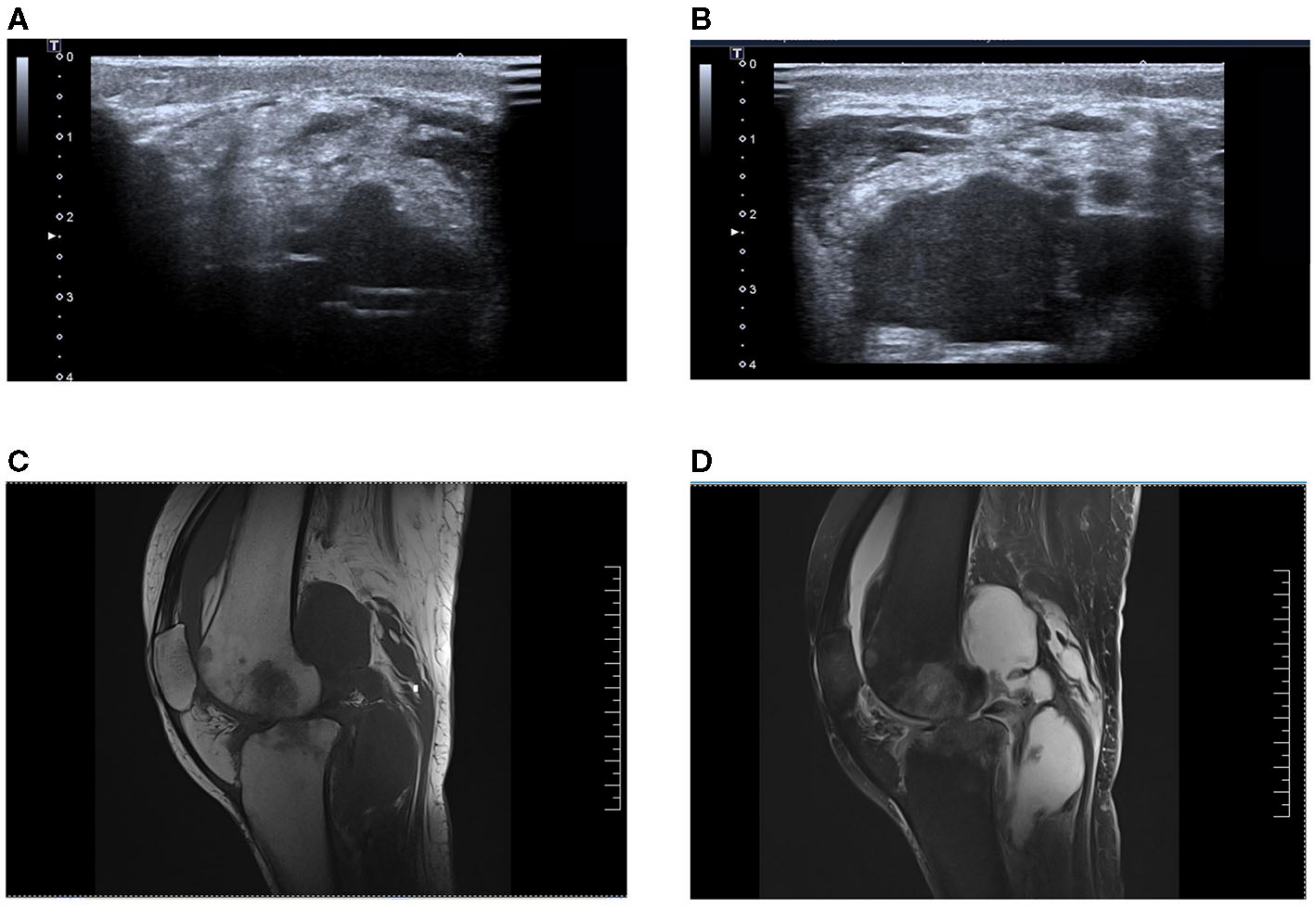
**(A,B)** Bedside ultrasonographic examination of knee joint cavity effusion in this patient. **(C,D)** Magnetic resonance imaging of the patient's knee cavity effusion, T1 image and T2 image, respectively.

After the patient was transferred to ICU, joint cavity pus was also taken for culture in addition to blood culture samples (ultrasound: Figures 2A,B, MRI: Figures 2C,D). Considering that the patient had negative culture multiple times in the previous month, the NGS examination was conducted for joint cavity pus and blood samples at the same time. Subsequently, NGS results indicated that the pathogen was “*M. tuberculosis*,” with acid-fast bacilli unexpectedly visible in articular cavity effusion. After an inquiry made about her medical history, it was known that the patient had a history of contact with pulmonary tuberculosis, and such symptoms as night sweating, coughing and expectoration were manifested since around the last month. Thus, the septic shock associated with *M. tuberculosis* was suspected. Five combinations of anti-tuberculosis drugs (HRZEO: Isoniazid, Rifampicin, Pyrazinamide, Ethambutol, Ofloxacin) were prescribed, while imipenem/cilastatin was downgraded to piperacillin/tazobactam and vitamin B6 was prescribed as a supplement. The bronchoalveolar lavage fluid was found negative for acid-fast staining. The T-SPOT results of the patients remained negative. The T lymphocyte count of the patient was shown to be extremely low. After about 10 days of anti-tuberculosis treatment, the body temperature improved, the ST segment of ECG decreased, myocardial enzymes returned to normal, shock was alleviated, and the count of platelets increased. Unfortunately, liver function deteriorated for the patient gradually. Five days after the adjustment made to anti-tuberculosis drugs, the patients showed such tuberculosis poisoning symptoms as high fever and shock, norepinephrine of 1.2 ug/kg/kg·min, which was accompanied by a decline in the count of platelet again. Afterwards, both liver function and cardiac function further deteriorated. Re-examination of blood NGS remained suggestive of *M. tuberculosis*. Our advice against further treatment was rejected by her family members.

## Discussion

In this study, a rare case of septic shock caused by the tuberculosis of the knee joint is reported. Sepsis puts a huge strain on global health care, and the severity of septicemia can affect the prognosis. For sepsis patients, the in-hospital mortality rate could exceed 10%, while the in-hospital mortality rate for the patients with septic shock and their serum lactate levels higher than 2 mmol/L could reach above 40% (3, 6–8). Additionally, there have been a large number of studies substantiating that the tuberculosis sepsis patients needing ICU treatment are at increased risk of in-hospital mortality (9–11). In one study, it was demonstrated that the previous medication on any antibiotics can cause delay to the diagnosis of *M. tuberculosis* (12). The latest evidence has been obtained to suggest that the delayed diagnosis of sepsis and late administration of antibiotics can increase the risk of mortality (13–16). In previous reports, the manifestation of *M. tuberculosis* septic shock was suggested as similar to that of bacterial septic shock, with early and appropriate antimicrobial treatment seemingly effective in reducing the risk of mortality (17). However, it is believed in this study that given the current routine bacterial culture of blood and other tissue samples, it is often difficult to identify early-stage tuberculosis septic shock in the absence of typical pulmonary lesions (18). A late start of anti-tuberculosis therapy will lead to the poor clinical outcomes (17). In this respect, NGS can provide more compelling evidence required for clinical diagnosis and treatment (19). Our patients showed a tendency to have their vital signs improved after NGS reported *M. tuberculosis* bacteremia and the timely use of anti-tuberculosis drugs, indicating the effectiveness of treatment. Due to late diagnosis and various complications, however, it failed to achieve a satisfactory outcome.

Though tuberculosis progressed slowly in most cases, it can be manifested in the form of disseminated tuberculosis with visible systemic symptoms. Tuberculosis sepsis shock (TBSS) is frequent to occur in those adults with low immunity, and the incidence is lower in children than in adults (20). TBSS can lead to such similar symptoms as fever, shortness of breath and multiple organ dysfunction to sepsis (17), especially in the patients with low immunity. In some studies, it has been suggested that TBSS can cause hyponatremia and anemia (9, 21–23). In this case, not only did the aforementioned situation occur following *M. tuberculosis* bacteremia, it also triggered various similar symptoms to G-bacilli bloodstream infection, for example, thrombocytopenia, elevated procalcitonin, persistent high fever and septic cardiomyopathy. Allowing for this, it is speculated in our study that TBSS may make it more difficult for clinicians to make accurate diagnosis in time than previously thought. A timely identification of pathogens and an early targeted use of antibiotics can be effective in improving prognosis for patients. However, active tuberculosis has long been the challenge facing clinical diagnosis and treatment. Due to atypical clinical manifestations and extrapulmonary tuberculosis infection, it is more difficult to make accurate diagnosis.

Given the microbiological characteristics of *M. tuberculosis*, the traditional etiological examination is disadvantaged by low sensitivity and long cycle, which cannot meet the urgent clinical needs for rapid etiological diagnosis of active tuberculosis (18). For quite long, the diagnosis of tuberculosis has been reliant on traditional acid-fast staining smears and the culture techniques of *M. tuberculosis*. Although culture remains the gold standard for the diagnosis of tuberculosis, it can not meet the requirements of rapid clinical diagnosis. In recent years, there have been a variety of new diagnostic techniques and methods for tuberculosis proposed. The emergence of molecular diagnosis technology in recent years achieves an important breakthrough, as represented by Xpert MTB/RIF detection kit (24, 25). WHO strongly recommends Xpert MTB/RIF for the preliminary screening of suspected drug-resistant tuberculosis among both adults and children. Besides, for such extrapulmonary specimens as cerebrospinal fluid, lymph nodes and other tissues derived from the patients suspected of extrapulmonary tuberculosis. Since its specificity exceeds 95%, it is applicable to the diagnostic test intended for tuberculosis. However, the sensitivity of both culture and Xpert MTB/RIF remains very low for the diagnosis of extrapulmonary tuberculosis. As a new laboratory method, next-generation sequencing (NGS) has been reported to be applied to assist the diagnosis of various pathogens, and in active pulmonary tuberculosis (19, 26). Plenty of studies have confirmed that NGS is quick to detect *M. tuberculosis* complex in various samples, with its sensitivity and specificity similar to Xpert MTB/RIF (5). The process of NGS detection mainly includes two parts: experimental operation (wet experiment) and bioinformatics analysis (dry experiment). Wet experiment mainly involves the following four steps: sample pre-processing, nucleic acid extraction, library construction and computer sequencing. Bioinformatics analysis mainly involves the following steps: data quality control, human sequence removal, microbial species comparison identification, drug resistance gene and virulence gene analysis. In recent years, the cost of NGS has been reduced to some extent, thus making it affordable for most families of ordinary patients. Compared with the huge amount of medical costs, actively defining the etiological basis and targeted medication can reduce the cost of treatment more significantly. Also, for patients with sepsis, besides *M. tuberculosis*, NGS can also be applied to screen other suspected pathogens fast, which is more beneficial to patients.

The clinical symptoms of tuberculosis exhibited by the patients with HIV-induced immunosuppression are different from those of immune hosts (27). Those infected with HIV are at increased risk to develop extrapulmonary tuberculosis compared to HIV-negative people. Those with less CD4 are more likely to develop extrapulmonary tuberculosis (28). The patients infected with tuberculosis and showing a small number of CD4 can show atypical chest imaging findings, and even normal chest imaging findings (29). In the relevant literature, it has been shown that in the case of immune disorders and destruction of the immune system, the T lymphocyte subset count is extremely low and the false negative rate of T-SPOT is fairly high, thus increasing the difficulty in making clinical diagnosis of *M. tuberculosis* infection. These atypical chest imaging examinations combined with atypical clinical manifestations could hinder the effort on early administration of anti-tuberculosis drugs, thus resulting in the poor prognosis of TBSS. This patient with anti-synthase syndrome was receiving immunosuppressant and hormone therapy for a long time. Consequently, the CD4 count was extremely low, as was the level of immunity. In case of infection with tuberculosis, the clinical signs of atypical extrapulmonary tuberculosis similar to those of patients with HIV infection would be manifested.

TBSS is extremely rare and there are only sporadic cases reported to date (9). The Centers for Disease Control and Prevention reported a decline in TB diagnosis. The rarity of TBSS makes this series of cases novel. According to our and previous cases, it can be found out that the case fatality rate of TBSS remains very high, with late diagnosis and delayed treatment being often contrary to the general epidemiological expectations of sepsis. The epidemic characteristics of tuberculosis vary in different countries and regions (2), so that each institution is supposed to determine its tuberculosis risk level according to the geographical location, the incidence of tuberculosis and the temporal trend of drug resistance. NGS is highly sensitive and rapid in the diagnosis of infectious pathogens, for which it is considered to be an important auxiliary diagnostic method for rare pathogens (5). Also, for those critically ill patients or the patients with unidentified pathogens, the effect of empirical treatment is not as satisfactory as expected, NGS can help identify the pathogen fast, for targeted anti-infective treatment to be carried out, which is of much significance to clinical treatment.

## Conclusion

Though the epidemic characteristics of tuberculosis vary by region, the high mortality rate and high missed diagnosis rate of TBSS remain common. Early diagnosis and early anti-tuberculosis treatment can be effective in improving the mortality of TBSS patients. Anti-tuberculosis treatment can be carried out directly in those high prevalence areas or for the patients with typical clinical manifestations. In this study, it is suggested that the application of NGS in sepsis patients can not only help clinicians identify the pathogen rapidly, but also screen rare pathogens, which is conducive to further reducing the mortality caused by sepsis.

## Data Availability Statement

The original contributions presented in the study are included in the article/supplementary material, further inquiries can be directed to the corresponding author/s.

## Ethics Statement

This study has been approved by the Scientific Research and Clinical Trial Ethics Committee of the First Affiliated Hospital of Zhengzhou University (Code 2019-KY-330). The patients/participants provided their written informed consent to participate in this study. Written informed consent was not obtained from the individual(s) for the publication of any potentially identifiable images or data included in this article.

## Author Contributions

LS and ZY were responsible for managing the patient. ZY collected data. ZW, HL, and TS revised the article. All authors have read and approved the final manuscript and contributed substantially to the work presented in this article.

## Conflict of Interest

The authors declare that the research was conducted in the absence of any commercial or financial relationships that could be construed as a potential conflict of interest.
